# Coercion in intensive care, an insufficiently explored issue—a scoping review of qualitative narratives of patient’s experiences

**DOI:** 10.1177/17511437221091051

**Published:** 2022-04-18

**Authors:** Susanne Joebges, Corine Mouton-Dorey, Bara Ricou, Nikola Biller-Andorno

**Affiliations:** 1 Clinic for Anesthesiology, Surgical Intensive Care Medicine and Postoperative Pain Therapy, Dortmund, Germany; 2 Institute of Biomedical Ethics and History of Medicine, 27217 University of Zurich, Zürich, Switzerland; 3 Department of Anaesthesiology, Pharmacology and Surgery Intensive Care, 27212 University of Geneva, Geneva, Switzerland

**Keywords:** coercion, ethics, intensive care unit (ICU), patient experiences, restraint

## Abstract

**Purpose:**

The use of coercion, in a clinical context as imposing a measure against a patient’s opposition or declared will, can occur in various forms in intensive care units (ICU). One prime example of a formal coercive measure in the ICU is the use of restraints, which are applied for patients’ own safety. Through a database search, we sought to evaluate patient experiences related to coercive measures.

**Results:**

For this scoping review, clinical databases were searched for qualitative studies. A total of nine were identified that fulfilled the inclusion and the CASP criteria. Common themes emerging from the studies on patient experiences included communication issues, delirium, and emotional reactions. Statements from patients revealed feelings of compromised autonomy and dignity that came with a loss of control. Physical restraints were only one concrete manifestation of formal coercion as perceived by patients in the ICU setting.

**Conclusion:**

There are few qualitative studies focusing on patient experiences of formal coercive measures in the ICU. In addition to the experience of restricted physical movement, the perception of loss of control, loss of dignity, and loss of autonomy suggests that restraining measures are just one element in a setting that may be perceived as informal coercion.

## Introduction

Understanding the experiences of patients in the intensive care unit (ICU) is important to tailor interventions that minimize hardship and suffering. When a patient is faced with potentially fatal health conditions that require life sustaining therapies such as invasive machines and powerful medication, they often are inevitably exposed to physical pain, psychological distress, and delirium.^1,2^ Patients find themselves in a situation of psychological and physical dependence on their caregivers, which can threaten their identity and dignity. Constraining the patient using an active or passive approach has been shown to increase the patient’s sense of dependency and suffering.^2^ When the ICU clinical teams support patients in understanding and adjusting to the situation, a sense of dignity and humanity can be restored.^3,4^

Coercion can be defined as “a mode of influence that operates by threats and force; aims at controlling the recipient’s being, movement, or will; and leaves, at least initially, its recipient *disadvantaged*.”^5^ In cases of coercion, a patient's liberty is restricted even if the patient is unable to actively recognize that he or she has been put under restraint.^6^ “The transition to coercion occurs where support for the patient’s self-determined formation of will ceases and the will of those treating the patient gains the upper hand without sufficient participation by the person concerned.”^6^

Using this broad definition of formal and informal coercion in a medical context, various situations in the ICU can be perceived by patients as coercion. In the ICU, restraints can take many forms; restraints are defined as, “something that limits an individual’s freedom of movement”^7^ and can include mechanical/physical,^8^ pharmacological, or psychological measures to actively or passively restrain the patient.^7,9^ The need for continuous monitoring of a patient can also be perceived as a kind of environmental restraint.^7,10^ Examples of informal coercion include threats, misinformation, manipulation, withholding privileges, and other influences.^9,11^ The various forms of formal and informal coercion are often used in combination with one another^12^.

The scientific literature mainly discusses the health professionals’ point of view, especially on physical restraints. One common justification for the use of both mechanical and pharmacological restraints is to protect the patient, for example, from injuries caused by unintentional removal of an endotracheal tube, cannula, catheter, or other devices.^13,14^ However, studies suggest that the incidences of unintentionally removed devices is even higher in restrained patients.^15,16^ Some studies report the use of physical restraint being involved in severe injuries and even death.^14,17^ In addition, physical restraint may increase the need for sedation, magnifying the risk of possible consequences that are associated with deeper sedation. There is an ongoing debate regarding the association of restraints with post-traumatic stress disorder (PTSD).^18^

The ambiguity around when and how to appropriately apply restraints is evident in the variation of their use and regulation in different countries. One study comparing the frequency of use of physical restraints in different countries showed a range of 7–87% of patients reporting the use of physical restraints of any form during their ICU stay.^19^ The PRICE study, conducted in 34 European ICUs in nine countries, found that an average of 33% of all patients were restrained in some way during their ICU stay.^12^ Some differences between countries were significant; in the United Kingdom and in Portugal, no patients were restrained, whereas, in Italy, every ventilated patient was restrained.^12^

Faced with these different approaches to coercive measures, and the ambiguity of their justification by different ICU teams there, is a need to broaden the scope of research on formal and informal coercion and include the perspective of patients to hopefully support the creation of guidelines that center the patient’s experience.

The aim of our narrative review was to understand how patients experience coercion and various forms of coercion in the ICU.

## Methods

With support from the University Library of Zurich, a review protocol for a scoping review on the topic of patient experiences with coercion in ICUs was created. Using a research protocol (PRISMA checklist 2018^20^), a search was run that included qualitative and mixed methods studies of patient experiences of restraints or other coercive measures in the ICU that were available up until August 2019. Reviews were assessed for qualitative statements. Based on the language skills studies in German and English were included.

Studies were excluded if they only relied on a quantitative approach because such studies do not allow patients to express their full range of experiences. As the aim was to gain insight into the patient’s perspective, records dealing with the experiences of nurses, physicians, and other clinical staff were excluded (Table 1).

**Table 1.** Inclusion/exclusion criteria.

inclusion criteria	Exclusion criteria
• 18 years old	• Setting psychiatric, geriatric, regular ward
• Mentioning restraining or coercive measures	
• Interviews conducted after stay in the ICU (patients)	• Experiences/case reports by clinical staff dealing with restraint patients
• qualitative studies and mixed studies	• Quantitative studies without qualitative component
• Reviews—with patient experiences in the ICU to find qualitative studies with coercive measures or restraint	• Questionnaire-only studies

The main search was run using subject heading/MeSH terms and a keyword search. The search strategy was adjusted for the individual interface of seven databases (PubMED, EMBASE, PsychINFO, Web of Science Core Collection, CINAHL, Scopus, and Cochrane Library). A Google search of gray literature was conducted, but no relevant studies were identified.

Most records were identified through the Scopus database (*n* = 193). Search terms are displayed in Table 2. Two examples of the search strategies are presented in Supplemental Appendix 1. On the first attempt, only four records were identified through PubMED. The terms were amended to include all patient experiences in the ICU and the search rerun. The search was conducted, including screening and abstract assessing, by two colleagues. Studies had to fit the inclusion criteria and full text had to be available. After the screening process, 17 studies remained. The quality of the included studies was assessed using the Critical Appraisal Skills Program for qualitative research (CASP).^21^ Authors have a medical background and further education in bioethics The STEPwise approach was used for data extraction.^22^ First, all relevant articles were searched for mentioning “coercion” or “restraints.” Secondly, in the text analysis, categories and themes were identified through discussion with the second researcher.^23^

**Table 2.** Search terms.

	Concept	Search terms
1	Coercion/restraints	Restraint
		Constraint
		Repress*
		Coercive
—	—	AND
2	ICU	Intensive care
		Critical care
		Critically ill
		Intensive care medicine
—	—	AND
3	Experiences	Experienc*
		Memor*
		Patient`s attitude
		Interview
		Narrative
		Qualitative trial
		Qualitative study
		Reviews
		Mixed method
—	—	NOT
4		Child*
		Psychiatr*
		Geriatric
		Experiences staff
		Quantitative survey only

## Results

**Figure 1.**
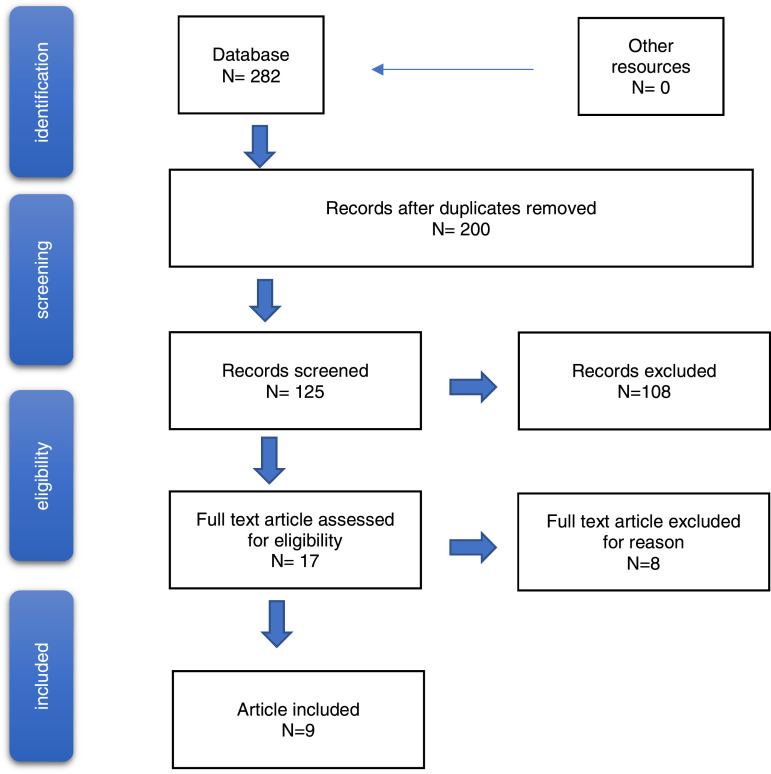
Graphical abstract.

After removing duplicates, abstract screening, and full text analysis, nine qualitative studies remained (Figure 1 + Supplemental Appendix 2: Table 1). One study (Minnick) dealt with the topic “physical restraint in intensive care units.” Eight studies covered communication, delirium, memories, and psychological experiences in intensive care units. In these eight studies, spare statements about coercion experiences were mainly related to physical restraint. Two of the studies were secondary analyses of previous study results.^24,25^ Themes and results on coercive measures/restraints regarding patient experiences can be found in Table 3.

**Table 3.** Themes paper—included.

Author	Themes	Experiences on coercion and restraining measures in the ICU
Minnick^31^ 2001/USA	Physical restraint	Patients do not remember great distress being restraint
Dziadzko^30^ 2017/USA	Acute psychological trauma in the critical ill	Procedures/restraint (24%) = “things that made patient feel worse”
Guttormson^24^ 2014/USA	Communication during mechanical ventilation	Accepting restrain when informed
Darbyshire^25^ 2016/Great britain	Delusion on intensive care	Restraint, restriction of movement by environment → lack of control
Moser^27^ 2018/Switzerland	Fear on chronical ill patients on ICU	Restraint (“wie eingeschlossen”), angst durch fixierung angst andere wirklichkeit: (“I felt trapped”)
Chahraoui^32^ 2015/France	Psychological experiences patients on ICU	Negative memories: Restraint
Russell^26^ 1999/Australia	Experiences in intensive care unit	Between delusion and reality: Restrain: “Feeling captive”
Clukey^28^ 2014/USA	Pain in intubated and sedated patients	Some lack of memory restraint intubation worse than restraint
Roberts^29^ 2019/USA	Experiences of acute mechanical ventilation	Restraint: “..Frightened and disconcerted and really anxious…”
		“I had no control”

The findings can be summarized into four main groups of patient experiences:

1. Patients’ memories are influenced by the critical illness, sedation, and delirium, but are real for the patient in the long term.

2. Statements exist that can be correlated with the perception of coercion and restraints.

3. The loss of dignity, dependence and discomfort can be perceived as a form of coercion by the patient.

4. Constructive communication can have a positive effect on patients’ experiences in the ICU.

1. Patients’ memories are influenced by the critical illness, sedation, and delirium, but are real for the patient in the long term.

Several studies reported that patients had incomplete, confusing, or delirious memories.^24-30^ Minnick et al. describe incomplete recollection in 60% of patients who underwent restraining measures during their stay in the ICU.^31^ In a study conducted by Chahraoui et al., all the participating patients initially reported having no memory of their ICU treatment.^32^ This might be explained by the severe and in some cases critical condition of patients included in the study. Moreover, metabolic, physiological, and psychological changes that affect memory can appear alongside multiorgan-related illnesses and can be worsened by sedative and/or analgesic drugs.^26,32^

**Figure 2.**
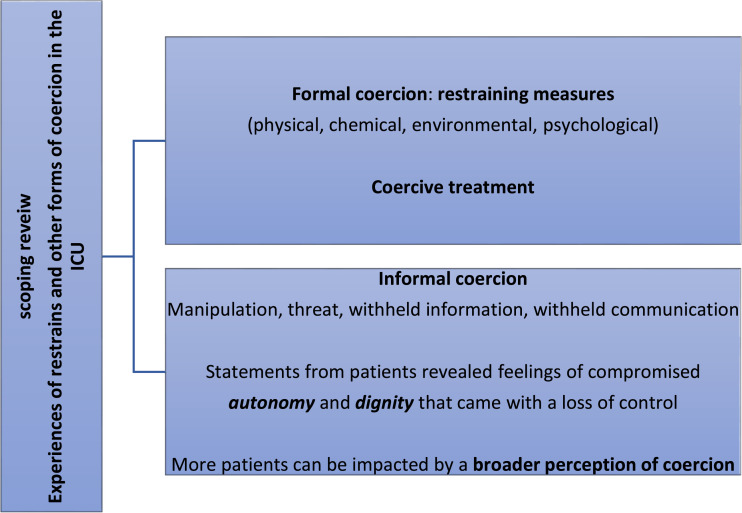
Flowchart (PRISMA).

The memories described, which were still present weeks later, represent a part of the patient’s subjective reality and may contain clues to the patient’s perception of coercion (Figure 2).

2. Statements exist that can be correlated with the perception of coercion and restraints.

Stories of being held down, restrained, and medicated are often embedded in findings on hallucination and distorted memories.^26,30,31^ Receiving life sustaining therapies in the ICU and including restrictions of movement often result in the feeling of being “locked up.“^27^ In a study by Chahraoui et al.,^32^ patients also had some recall of physical restraint, “painful memories of being tied down,” after memories were triggered by an external source.^32^ Furthermore, restrictions by equipment often contributed to the perception of being restrained. Darbyshire et al. (2016) describe a wide range of perceptions and delusions that include abduction, imprisonment and “feelings of restraint” generated by “restriction of movement by the equipment” and “blurred reality”.^25^ In the collection of patient experiences of acute mechanical ventilation reported by Roberts et al., most patients had memory of mechanical ventilation. They associated restraining measures and mechanical ventilation with fear and helplessness.^29^ When patients were able to recall their ICU stay and experience with restraints, they expressed a range of negative feelings, which could be related to some forms of restraint.

3. The loss of dignity, dependence and discomfort can be perceived as informal coercion by the patient

Table 4 shows that negative experiences in the ICU were not only related to the use of physical restraints; other aspects commonly linked to negative perceptions included lack of control, helplessness, noise, anxiety, pain, ventilation, dependency, insomnia, and delirium with or without accompanying hallucinations. Endotracheal tubes, which interfere with active communication during ventilation, were especially linked to the perception of helplessness and dependency.^24-30,32^ Many patients attributed a sense of loss of dignity to dependency and lack of control.^25-27,29^ Moreover, feelings like powerlessness, uncertainty, and “feeling lost” were commonly described in combination with anxiety.^27^ Dziadzko et al. reported that restraining measures worsened the emotional state of the patient. Indeed, in their study, “The inability to communicate (34%), environmental factors (noise, alarms, laughter) (30%), procedures and restraints (24%), and intubation (12%)” were listed as the primary factors leading to negative emotions and worsened the psychological stress that is already present in the ICU.^30^ These difficult experiences suggest that patients in the ICU are especially vulnerable to feeling or being informal coerced during an experience defined by a sense of loss of autonomy, identity, and dignity. These situations carry the risk that the will of those “treating the patient could gain the upper hand without sufficient participation by the person concerned (Figure 2).”^6^

4. Constructive communication can have a positive effect on patients’ experiences in the ICU

**Table 4.** negative/helpful experiences on ICU.

	Negative experiences	Helpful experiences
**Minnick**	- Delirium	- Communication
	- Intubation	
**Dziadzko**	- Inability to communicate	- Communication
	- Environmental factors	- Support by family
	- Procedures and ***restraints***	- Emotional support
	- Intubation	- Moral support
**Guttormson**	- Lack of information → *helpless*	- Benefits of information/ommunication → relieve anxiety and “settle the mind.”
	- Perceived lack of information was associated with *not feeling in control and helplessness*	- Information helped tolerate treatments including the ventilator and ***physical restraints***
	- Cannot communicate	
**Darbyshire**	- Delusion	
	**- *Restraint*,** restriction of movement by equipment → lack of control	
	- Loss of autonomy	
	- Isolation	
**Moser**	- Fear by delusions—nightmares helpless/dependency without dignity (feeling like an object)	- Information
		- Trust in people
**Chahraoui**	- Lack or false memory	- Positive memories: Support from the health care team and family
	- Negative memories related to ***physical restraint***, sleep disorders difficulties arising from not being able to talk, pain, feelings of incomprehension about ICU stay, feelings of fear, sensation of impending death and a feeling of having been abandoned by their family/caregivers	
**Russell**	- Lack of communication	- Presence of good communication
	- Delusion: Stories of being held down, ***restrained*** and medicated ‘into oblivion'	
	- Poor communication	
	- Lack of privacy, fear, pain, and noise	
	- Lack of dignity	
**Clukey**	- Intubation worse than ***restraints***	- Nurses provide information and anticipatory guidance helped relieve anxiety for both
		- Good communication
**Roberts**	- Lack of communication	- Effective communication
	- Lack of control	
	- Frightened and disconcerted, anxious	
	- Lack of control	

ICU: Intensive Care Unit; CASP: the Critical Appraisal Skills Program for qualitative research.

In contrast to other reports, the studies by Clukey et al. and Minnick reported patients who recalled their stay in the ICU and restraining measures without negative associations. Some patients were able to understand the use of restraint as for ‘*their own good*’ if communicated and explained appropriately.^28,31^ Likewise, Guttormson et al. described experiences by patients where information was helpful in the acceptance of procedures and restraints.^24^ For some patients, the presence of family members ameliorated some of the challenging factors of their ICU stay.^28,30,32^ All studies stated that information and good communication could reduce stress or fear and be beneficial for patients in the ICU, whether the source of information came from family or providers (Table 4). Patients and relatives both described positive memories of emotional and moral support from the care team that improved the overall experience.^30^ Good communication and understanding the reasons for the restraints alongside family and staff emotional support seemed to play a critical role in the patient’s acceptance and ability to process the restraints.

## Discussion

Patients’ memories are influenced by the critical illness, sedation, and delirium, but remain real for the patient over time. When reflecting on the overall experience of being in the ICU, physical restraints were perceived as a part of a threatening situation and as going against the patient’s free will. Additionally, the loss of dignity, dependence, and discomfort can be perceived as informal coercion by the patient. Informal coercion can be achieved by withholding communication and information. Constructive communication and positive interaction between the patient and the team can be helpful for the patient’s wellbeing in the critical care settings.

Autonomy and dignity are universally recognized as ethical principles to respect, even though their specifications may vary in national laws.^33,34^ Coercion can arise by way of measures that restrict freedom (restraints). There is widespread consensus that restraint should only be applied in exceptional circumstances and if communicated clearly.^16,35,36^ Even though restraints are used to protect the patient from self-induced harm, there is limited evidence that physical and/or pharmacological restraints are related to better outcomes.^14,37^ The intensive care community is aware of the psychological and ethical risks of restraining measures.^16^ To avoid restraint in the ICU, guidelines are suggested, and training efforts have been established.^38,39^ It is valuable to have the patient’s perspective on restraining measures when developing such guidelines and messaging.^40^

Individual freedom may be limited by situations in which one cannot express oneself, cannot pursue one's individual goals, or cannot access opportunities to act.^41^ There is also a perception in the literature of patients’ experiences regarding lack or bad communication, lack of information, lack of control, helplessness, anxiety, pain, ventilation, dependency, insomnia, and delirium that carry a risk of informal coercion and overriding the autonomy and dignity of the individual.^42,43^ Moreover, there remains an asymmetrical power dynamic between the clinical team and the patient.

Some authors argue that non-pharmacological strategies should be considered before using restraints.^38^ Other studies have suggested that allowing the patient to remain awake; mobilizing them; administering sufficient therapy for pain, delirium, and anxiety; and including the family as much as possible in the process are all useful.^38,39^ Here, the challenge seems to be how best to keep patients awake and mobile during their stay in the ICU to improve quality of life.^44^

When adequate and focused communication is provided, some patients can accept restraining measures for their own good. This is a challenge, though, as patients in the ICU are often incompetent or agitated. Communication in such situations is difficult. Patients do report feeling understood and supported when talk to.^28,31^ Patients want to be informed and updated and want to discuss problems to be a partner in decision-making.^45^ Allowing family members to be with the patient in the ICU helps to empower patients and has been shown to positively influence outcomes and decrease length of stay.^46,47^ To preserve a sense of reality and to provide the best possible support, patient diaries, a later visit to the ICU, or psychological support post-discharge may be useful.^48,49^

In this research, the identified experiences of coercion in the ICU were not only related to the use of restraints. The perception of helplessness, dependency, powerlessness, and the loss of dignity may additionally be considered as a risk of disadvantage and informal coercion.

Guidance and education on how to recognize the patient as a person and communicate with him/her as a partner may reduce the perception of coercion and related negative emotions. Following a care ethics approach, a patient’s perspective and participation should be included in the therapeutic journey.^40^

### Limitations

A major limitation of the present narrative review is the lack of recollection of ICU experiences by patients, but they were real for the patients. This has been widely reported by previous studies and can be difficult to avoid as it is related to the actual clinical situations of ICU patients. Another limitation concerns language. The narrative review only included studies available in English and in German, and may, therefore, have missed important contributions published in other languages.

## Conclusion

Few qualitative studies have been performed on patients’ experiences of formal coercive measures in the ICU. Patients’ memories are influenced by the critical illness, sedation, and delirium, but remain real for the patient over time. Restraining measures can cause a range of negative emotions. In addition to the experience of restricted physical movement that restraining measures cause, the perception of loss of control, loss of dignity, and loss of autonomy may lead to a broader perception of informal coercion. By withholding communication and information a situation may be experienced by the patient that can be classified as informal coercion. Health professionals may underestimate how deeply patients are affected by the perception of formal and informal coercion. It may be relevant to broaden the view from physical or chemical restraining measures in the ICU to include these psychological vulnerabilities and the risk of informal coercion. More research on patient experiences is needed to reach a more comprehensive understanding of how patients perceive coercion in the ICU and to identify opportunities for further improvement of intensive care.

## Supplemental Material

sj-pdf-1-inc-10.1177_17511437221091051 – Supplemental Material for Coercion in intensive care, an insufficiently explored issue—a scoping review of qualitative narratives of patient’s experiences Click here for additional data file. Supplemental Material, sj-pdf-1-inc-10.1177_17511437221091051 for Coercion in intensive care, an insufficiently explored issue—a scoping review of qualitative narratives of patient’s experiences by Susanne Joebges, Corine Mouton-Dorey, Bara Ricou and Nikola Biller-Andorno in Journal of the Intensive Care Society
