# Supplementary material for: Coercion in intensive care, an insufficiently explored issue—a scoping review of qualitative narratives of patient’s experiences
Source: J Intensive Care Soc. 2022 Apr 18;24(1):96–103. doi: 10.1177/17511437221091051 (PMC9975803; doi:10.1177/17511437221091051)
Supplement: sj-pdf-1-inc-10.1177_17511437221091051 – Supplemental Material for Coercion in intensive care, an insufficiently explored issue—a scoping review of qualitative narratives of patient’s experiences [file sj-pdf-1-inc-10.1177_17511437221091051.pdf]

Appendix 1: *exemplary* search strategies Scoping Review

Scopus
TITLE-ABS-KEY(coercive OR restraint OR constraint OR bond OR repress*) AND TITLE-ABS-KEY((intensive OR critical) W/3 (care OR ill* OR medi?in*)) AND TITLE-ABS-KEY(experienc* OR "to undergo" OR memor* OR «patients attitude») OR ((interview OR narrative OR qualitative) W/3 (trial OR study)) AND NOT TITLE-ABS-KEY (psychiat*) AND NOT TITLE-ABS-KEY (child* OR infant* OR newborn OR p?diatr*) AND NOT TITLE-ABS-KEY(nurs*)
(N=193)

Pubmed/Medline
(((((((("Restraint, Physical"[Mesh] OR "Coercion"[Mesh])) AND ("Critical Care"[Mesh]) OR "Intensive Care Units"[Mesh])) AND (coercive[Title/Abstract] OR restraint[Title/Abstract] OR constraint[Title/Abstract] OR bond[Title/Abstract] OR repress[Title/Abstract])) AND (intensive care[Title/Abstract] OR critical care[Title/Abstract] OR critical ill*[Title/Abstract] OR ICU[Title/Abstract])) AND ("patient experience"[Title/Abstract] OR "to undergo"[Title/Abstract] OR memor*[Title/Abstract] OR «patients attitude»[Title/Abstract] OR interview[Title/Abstract] OR narrative[Title/Abstract] OR qualitative trial[Title/Abstract] OR qualitative study[Title/Abstract]))) NOT psychiat*[Title/Abstract]) NOT nurs*[Title/Abstract]) NOT (child* [Title/Abstract] OR infant* [Title/Abstract] OR newborn [Title/Abstract] OR p?diatr*[Title/Abstract])). (N=4)
Advanced search criteria
PubMed
("patient experience"[Title/Abstract]) AND ("intensive care"[Title/Abstract] OR ICU[Title/Abstract] OR "critical ill"[Title/Abstract] OR "critical care"[Title/Abstract]) (N=100, included= 38)

Appendix 2:

Table 1: criteria of the included studies

**author**	**method**	**timing interview**	**ICU**	**patient - ICU criteria**	**Patients/ family**
**Minnick**	semi-structured interview	before discharge	medical/surgical		15
**Dziadzko**	structured interview	72h post ICU	medical/surgical	> 48h ventilation	50/44
**Guttormson**	structured interview	to18 month second analysis	medical/surgical	Median >72h ventilation	31
**Darbyshire**	interview	second analysis			40/37
**Moser**	interview	to 35d post ICU	interdisciplinary ICU	>14 d ICU, >8d ventilation	7
**Chahraoui**	interview + questionnaire	3 months after ICU			20
**Russell**	interview + questionnaire	6 months after discharge			86 interviews 298 questionnaires
**Clukey**	Semi-structured interview		medical	ventilated	14
**Roberts**	quantitative + qualitative	before discharge	medical/surgical	ventilated in the ICU	130(quantitative) 15 (qualitative)

Author	Are the results value?	What are the results?	Will the results help locally
**Minnick** [1]<br>2001/USA	5x yes<br>1x can`t tell	2x yes<br>1x can`t tell	Overall situation leading to restraints is perceived stressful
**Dziadzko** [2]<br>2017/USA	5 x yes<br>1 x no	2x yes<br>1x can`t tell	Enlighten what relatives and family perceive in the ICU
**Guttormson** [3]<br>2014/USA	5x yes<br>1x can`t tell	3x yes	highlights the impact- through the patients' own words - of communication challenges during mechanical ventilation
**Darbyshire** [4]<br>2016/Great Britain	5x yes<br>1x can`t tell	3x yes	indicate that there are many more patients whose experience of the ICU could be improved with earlier support.
**Moser** [5]<br>2018/Switzerland	6x yes	3x yes	Study allows health professionals to gain insight into patient anxiety experiences
**Chahraoui** [6]<br>2015/France	5 x yes<br>1 x no	3x yes	qualitative interview-based approach, to improve the management of patients in routine practice
**Russell** [7]<br>1999/Australia	5x yes<br>1x can`t tell	3x yes	both technical expertise and empathy were needed in ICU
**Clukey** [8]<br>2014/USA	5x yes<br>1x can`t tell	3x yes	Evaluating the effectiveness of practice changes and the potential increase in positive outcomes
**Roberts** [9]<br>2019/USA	6x yes	3x yes	research into effective communication methods for critical care patients who are mechanically ventilated
